# Independent and Combined Associations of Blood Manganese, Cadmium and Lead Exposures with the Systemic Immune-Inflammation Index in Adults

**DOI:** 10.3390/toxics11080659

**Published:** 2023-08-01

**Authors:** Qiya Zhong, Wenxin Zhou, Jiaqi Lin, Wen Sun, Yao Qin, Xiang Li, Huadong Xu

**Affiliations:** 1 School of Public Health, Sun Yat-sen University, Guangzhou 510080, China; zhongqy33@mail2.sysu.edu.cn; 2 School of Public Health, Hangzhou Medical College, Hangzhou 310013, China; zzzhouwx@foxmail.com (W.Z.); linjiaqi1@foxmail.com (J.L.); 881012022125@hmc.edu.cn (W.S.); qin_yao@foxmail.com (Y.Q.); 3 School of Nursing, Yanbian University, Yanji 133000, China; 0000008757@ybu.edu.cn

**Keywords:** systemic immune-inflammation index, manganese, cadmium, lead, co-exposure, adults

## Abstract

Manganese (Mn), cadmium (Cd) and lead (Pb) have toxic effects on the immune system. However, their independent and combined effects on immune-inflammation responses are unclear. In recent years, the systemic immune-inflammation index (SII) has been developed as an integrated and novel inflammatory indicator. A retrospective cross-sectional study of 2174 adults ≥20 years old from the National Health and Nutrition Examination Survey (NHANES) 2015–2016 was conducted. Generalized linear models were used to evaluate the independent and combined associations of SII with blood Mn, Cd and Pb levels. As continuous variables, both blood Cd and Mn showed dose-dependent relationships with the SII before and after adjusting for all potential confounding factors. Metal concentrations were then converted into categorical variables. Compared with the adults in the lowest Cd or Mn tertile, those in the highest tertile had higher risks of elevated SII. Furthermore, co-exposure to Mn and Cd also showed a positive relationship with the SII after adjusting for all confounding factors. However, the single effect of Pb exposure and the joint effect of Pb and other metal exposures on the SII were not observed. This study provides important epidemiological evidence of the associations of SII with single and co-exposure effects of blood Mn, Cd, and Pb.

## 1. Introduction

Manganese (Mn), cadmium (Cd), and lead (Pb) are typical metals that can harm public health globally. Pb and Cd are generally present in batteries, pigments, medications, and other artificially produced goods [1] and have negative impacts on the immune system [2,3]. Exposure to Cd alone can damage the immune system by affecting innate cells, which changes T-cell production and function [4]. Previous research demonstrated that the combination of Cd and Pb could trigger inflammatory responses by releasing free radicals and reducing antioxidant defenses, which could lead to cell necrosis and the release of intracellular inflammatory chemicals [5]. Furthermore, children’s erythrocyte CD44 and CD58 expression may be compromised by prolonged exposure to Pb alone [6]. The U.S. Centers for Disease Control and Prevention (CDC) established safety guidelines for adults with blood Cd and Pb levels of 5 μg/L and 10 μg/L, respectively [7]. A systematic review of the literature proposed that Mn presented U-shaped exposure–response effects on health [8]. In a prior investigation, Mn was found to be essential for nutritional immunity at low concentrations [9], whereas too much Mn may raise the risk of immune toxicity in a variety of vertebrate species, including fish, mice and birds [10]. According to the results of previous animal studies, Mn can alter lymphocytes and other physiological variables as well as enhance susceptibility to infection on both the inner and exterior parts of the body [11,12,13,14]. The majority of research involving the effects of Mn on human health has focused on its neurologic impacts and found small neurologic abnormalities related to blood Mn levels [15]. Epidemiological research has linked high occupational exposure to Mn to a reduction in T-lymphocyte numbers, but only in adult men [16].

Several inflammatory biomarkers, such as neutrophil count, neutrophil to lymphocyte ratio (NLR) [17,18], monocyte to lymphocyte ratio (MLR) [19], and platelet to lymphocyte ratio (PLR) [19,20,21], have been utilized in previous studies to prognosis colorectal cancer and lung cancer [22,23]. These biomarkers, however, may not correctly indicate the inflammatory condition since they are only related to one or two types of immune-inflammatory cells. As a brand-new inflammatory biomarker that correlates with neutrophil, lymphocyte, and platelet counts [24], the systemic immune-inflammation index (SII) can be used to evaluate the prognosis of patients with cancer and coronary artery disease (CAD) [25,26,27]. Hence, SII was proposed to be treated as an accurate index to reflect inflammation status in humans [28].

Currently, most researchers have paid attention to the single effect of heavy metals on human health. Several studies explored the effects of co-exposure to Pb and Cd [29,30,31] and Pb and Mn [32,33] on human health. A few studies on animals revealed that rats exposed to Mn, Cd, and Pb at the same time were more likely to develop hypolipidemia [34]. However, no previous study specifically addressed the independent and combined effects of these three elements on SII. Therefore, we decided to examine the independent and combined associations of Mn, Cd and Pb with the SII in a nationally representative population.

## 2. Materials and Methods

### 2.1. Study Population

The data used in this study were collected from the National Health and Nutrition Examination Survey (NHANES) 2015–2016. The NHANES is a cross-sectional survey to assess the health and nutritional status of the U.S. population. Data were obtained through interviews, physical investigations, and laboratory tests [35]. The NHANES is a well-known public database that provides publicly available data for researchers around the world. To date, the data from NHANES have been widely used in studies to explore the impact of environmental pollutants on human health. The findings from the NHANES can provide a reference for other population studies as well as research clues for toxicological studies, even though the NHANES is an American database. The NHANES was reviewed and approved by the Ethics Review Board of the National Center for Health Statistics (NCHS). Each participant voluntarily provided their written informed consent. More details about this study are documented on the official website (https://www.cdc.gov/nchs/nhanes/, accessed on 29 March 2023).

**Figure 1:**
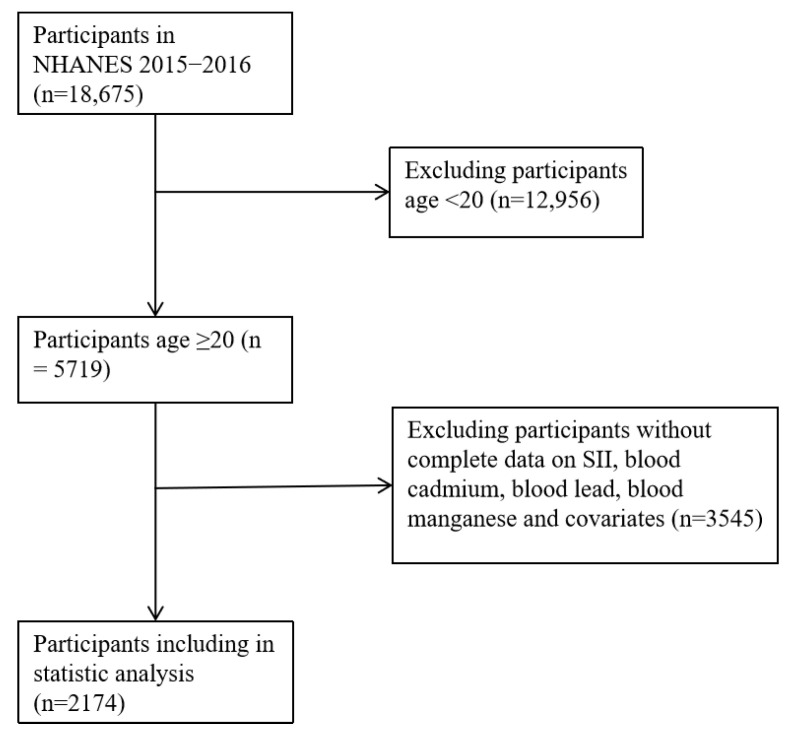
Flowchart for selection of the study participants.

After completing SII results and blood-heavy metal tests, participants under 20 years old were excluded. People who lacked important study-relevant variables were eliminated. Finally, 2174 participants were included in the analysis. Figure 1 displays the flow chart for the inclusion and exclusion of the study participants.

### 2.2. Blood Mn, Cd and Pb Measurements

In the NHANES project, one-half of a sample from participants aged 12 years and older were selected to measure the levels of Pb, Cd and Mn. Whole blood samples were obtained and stored at −30 °C, and then measured by the National Center for Environmental Health and the Centers for Disease Control and Prevention. Concentrations of Mn, Cd and Pb were detected in the whole blood samples using mass spectrometry after a simple dilution sample preparation step. More detailed information can be found in the laboratory manual [36]. The results of Mn, Cd and Pb were directly collected in this study.

The detection limits (LODs) for Pb, Cd and Mn were 0.07 µg/dL, 0.1 µg/L and 0.99 µg/L in NHANES 2015–2016, respectively. The concentrations in this study are in the same range as those reported in recent studies [7,31,37,38]. The concentrations of Mn, Cd and Pb below the LODs were substituted with the limit divided by $\sqrt{2}$ [7].

### 2.3. Peripheral Blood Cell Count Measurement

The platelet counts, neutrophil counts and lymphocyte counts in the blood samples were detected using a Coulter HMX Hematology Analyzer. The SII level was calculated following our previous paper (platelet count × neutrophil count/lymphocyte count) [24]. The LOD for SII was dependent on platelet count, which was 3.0 × 10^3^ cells/µL.

### 2.4. Covariates

Several variables were regarded as potential confounding factors in the analysis, according to earlier studies [39], including age, gender, body mass index (BMI), race/ethnicity, educational levels, and annual family income and lifestyle factors (alcohol intake and exposure to smoking). BMI was expressed in kg/m^2^ and further classified into three groups (<25, 25–30, and ≥30 kg/m^2^) [38]. Alcohol intake (yes/no) was defined on the label as whether you had at least 12 alcoholic drinks/year. Exposure to smoking (yes/no) was classified according to serum cotinine level (0.015 ng/mL) [40,41,42].

### 2.5. Statistical Analysis

SPSS (version 24.0, IBM Corp., Armonk, NY, USA) and R (vision 4.0.3, R Foundation for Statistical Computing, Vienna, Austria) were used to analyze the data. “MASS” and “ggplot2” packages were used in the R program. Differences in baseline characteristics were compared using the Mann–Whitney *U* test or the Kruskal–Wallis test. Spearman analysis was used to calculate the correlations of SII, age and BMI with the concentrations of blood Mn, Cd and Pb. Furthermore, the general linear model (GLM) was applied to assess regression coefficients (βs) and 95% confidence intervals (CIs) between SII and blood concentrations of Mn, Cd, and Pb as well as co-exposure effects of three elements. The GLM is a common method used as a numerical solution of ordinary differential equations [43]. The value of SII was taken as the dependent variable and the metal concentration was taken as the independent variable. In the multivariate regression analysis, metals were also sorted into tertiles as categorical variables. The effects of metal co-exposure on SII were examined using a combined multiplicative variable (Mn*Cd) which was widely applied to explore the combined effect in previous studies [43,44,45,46,47]. Finally, three models were available for analysis: Crude mode, no modification; Model I, adjusted for age, race/ethnicity, sex, and BMI; and Model II, adjusted for all variables in Model I plus additional adjustments for educational levels, annual family income, alcohol consumption, and exposure to smoking. Statistical significance was defined as a two-sided *p* value < 0.05.

## 3. Results

### 3.1. Characteristics of the Study Population

The baseline demographic information about the concentrations of blood Mn, Cd and Pb is presented in Table 1. Blood Mn levels were higher in younger adults, females, other races, non-drinkers and those with higher SII levels. Blood Cd levels were higher in older adults, females, non-Hispanic black and other races, and those with middle school educational levels, lower annual household income, lower BMI, or exposure to smoking. Blood Pb levels were higher in older adults, males, non-Hispanic black and other races, drinkers, those exposed to smoking, and those with middle school educational levels and lower BMI.

### 3.2. Correlations between Continuous Variables and Blood Concentrations of Mn, Cd and Pb

The results of the correlation analysis are presented in Table 2. Blood Mn had a positive correlation with SII and BMI (*r* = 0.075 and 0.045, respectively), but a negative correlation with age (*r* = −0.144). The Cd concentration was positively associated with SII (*r* = 0.055) and age (r = 0.076) but negatively associated with BMI (*r* = −0.093). Additionally, there was a positive correlation between blood Pb concentration and age (*r* = 0.286) but there was a negative correlation between Pb and BMI. In contrast, the results showed no significant correlation between blood Pb and SII (*r* = 0.090 and *p* = 0.629).

### 3.3. Associations of SII with Mn, Cd and Pb

**Figure 2:**
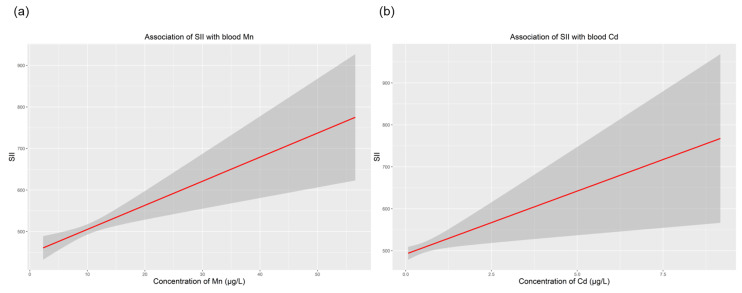
Dose–response relationship of SII with concentrations of blood Mn and Cd. (**a**) Association of SII with blood Mn; (**b**) association of SII with blood Cd. All models were adjusted for age, sex, race/ethnicity, BMI, education, annual family income, alcohol intake and exposure to smoking. Abbreviations: BMI, body mass index; SII, systemic immune-inflammation index.

Table 3 presents the associations of SII with blood Mn, Cd and Pb levels using generalized linear analyses. The concentrations of Mn and Cd, as continuous variables, were positively correlated with the SII (all *p* < 0.05). We found that for every 1 unit increase in blood Mn, the estimated *β* values indicated an increase of 5.80 in SII (95% CI: 2.53–9.06 and *p* = 0.001). For blood Cd, the estimated *β* value for increased SII was 30.06 (95% CI: 7.00–53.12 and *p* = 0.011). After concentrations of Mn and Cd were converted into categorical variables, individuals whose blood concentrations of Mn were in the third tertile (>10.94 μg/L) had a significantly higher SII (*β* = 33.49, 95% CI: 4.31–66.66, and *p* = 0.026) than those whose blood concentrations of Mn were in the first tertile (<8.21 μg/L). A similar pattern was also observed in the relationship between blood Cd and SII. The SII value of individuals in the third Cd tertile (>0.42 μg /L) was significantly higher (*β* = 48.91, 95% CI: 16.20–81.64, and *p* = 0.003) than that of individuals in the first Cd tertile (<0.22 μg/L). The dose–response relationship of SII with blood Cd and Mn was then separately explored via the R software (Figure 2). In contrast, there was no significant linear association between blood Pb levels and the SII. When blood Pb was treated as a continuous variable, the estimated *β* value of SII was 6.63 (95% CI: −4.13–17.40 and *p* = 0.227). After we adjusted all confounding factors and converted blood Pb to a categorical value, the estimated *β* was −1.31 (95% CI: −37.63–35.00 and *p* = 0.943).

### 3.4. The Relationship of SII with Co-Exposure to Blood Mn and Cd

**Figure 3:**
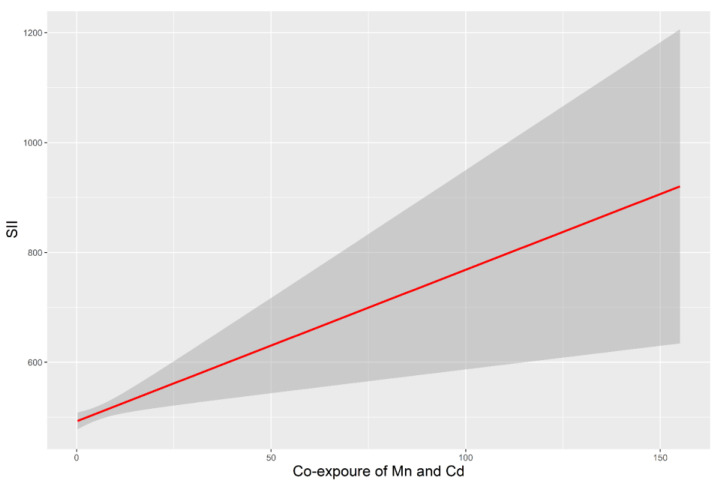
SII associated with co-exposure to Mn and Cd. All models were adjusted for age, sex, race/ethnicity, BMI, education level, annual family income, alcohol intake and exposure to smoking. Abbreviations: BMI, body mass index; SII, systemic immune-inflammation index.

The potential interactions of Mn and Cd are listed in Table 4. There was a positive correlation of SII with co-exposure to blood Mn and Cd (as a continuous variable) (*p* < 0.05). After transforming the co-exposure value of Mn and Cd into a categorical variable, adults in the third tertile had a higher SII than those in the first tertile. The model of the dose–response relationship of co-exposure to Cd and Mn with SII is shown in Figure 3. However, no significant interactions between Pb and the other two metals were observed.

## 4. Discussion

People are generally exposed to multiple heavy metal elements rather than a single metal element in the real world [46]. The interactions between heavy metals can be additive, synergistic, antagonistic, or independent, and can produce different biochemical changes in different regions of the human body [7,29,37,46]. However, most research has focused on the health risks of single heavy metal exposure, with little focus on the health effects of interactions between multiple heavy metals. It is not possible to clarify the interactive effects of combined heavy metal exposure. Therefore, combined exposure to heavy metals is a key gap in the study of population health risks, and it is necessary to explore the health effects of combined exposure to heavy metals.

Mn exposure in the general population is mostly connected with daily nutrition and water intake [48] as well as occupational [49] and environmental exposure [50]. Cd enters the human body due to the factors mentioned above in addition to tobacco use [51]. There is mounting evidence that exposure to Mn, Cd, and Pb alone or in combination has harmful consequences on human health, including neuroinflammation, cognitive impairment, and behavioral problems [45,52]. In an animal study, Pankaj and colleagues found that an oral dose of Mn and Cd could cause inflammation in mice, which results in a faster onset of viral infection in the brain [53].

Heavy metal exposure can increase the risk of immune system disorders, according to numerous studies [54]. SII, which is composed of neutrophils, lymphocytes, and platelets, is an immunological biomarker that is easily detected. The majority of white blood cells are neutrophils, which play an important role in triggering immunological responses and reducing chronic inflammation by producing neutrophil elastase [55]. Lymphocytes, which express more than 75% of the human genome, can control inflammation through the immune system [56]. The functions of platelets include activating innate and adaptive immune responses [57]. Therefore, the level of SII may reflect the inflammatory condition in the body [58]. High SII values are frequently accompanied by lymphopenia, neutropenia, or thrombocytosis [59]. According to our current findings, those with higher blood Mn and Cd levels as well as co-exposure to Mn and Cd had considerably higher SII levels. After adjusting for potential confounding variables, we found that the SII showed positive linear relationships with blood Mn, Cd levels, and co-exposure to Mn and Cd. In addition, metal exposure has greatly improved. Since the levels of metal exposure within the range of reference values were still associated with the immune system, these results may support the hypothesis that there might be a new threshold of metals for the immune system in humans. It is considered necessary to continue to monitor and study environmental metal exposure.

Pb exposure occurs in humans from several sources, including soil, food, dust, and products used in manufacturing in daily life or at work [60]. In addition, there is a complicated link between immune system health and Pb exposure. Mishra et al. reported that blood Pb levels above 25 g/dL had detrimental effects on the individual’s immune system by lowering the quantity and proportion of CD3+ and CD4+ cells [61]. In contrast, Dou et al. reported that there was no significant correlation between Pb and T-lymphocytes, B-lymphocytes, and other immune cells, as well as TH1/TH2/TH17 cytokines, while those exposed to high levels of Pb had higher percentages of CD3+ cells than those exposed to low levels of Pb [62]. However, in this study, we found that there were no linear relationships of SII with Pb exposure alone, co-exposure of Pb and Cd, and co-exposure of Pb and Mn as well as co-exposure of Pb, Mn, and Cd. A potential mechanism may be related to the effect of Pb on the immune system. A previous study showed that Pb exposure not only decreased lymphocyte numbers and suppressed the adaptive immune responses but also changed the innate immune response [63]. As a result, there was no linear relationship between Pb and SII. In the future, additional nonlinear studies on blood Pb and SII are needed. We did not observe the single effect of Pb exposure and the joint effect of Pb and other metal exposures on the SII, suggesting the limited effect of Pb levels observed in this study.

Our study had several strengths. First, only a few earlier investigations had examined the link between SII and exposure to heavy metals. This is the first study to demonstrate the effects of Mn, Cd, and their joint exposure on SII in humans. Second, a substantial connection of SII with blood Cd and Mn as well as co-exposure to Cd and Mn was supported by the large sample size. Additionally, this study accounted for potential confounding variables that might alter the relationship between blood element concentrations and SII using extensive covariate data.

However, there were some limitations to our study. First, the cross-sectional study design precluded us from drawing judgments about the cause. Instead, prospective studies are needed to fully understand how exposure to heavy metals affect the systemic immune-inflammatory condition. Furthermore, although we investigated the relationships between exposure to heavy metals and the SII, this study only explored one indicator of the immune system. More research is still necessary to ascertain the link between exposure to metals and additional immuno-inflammatory biomarkers, such as the NLR, PLR, and LMR. Furthermore, the data on neutrophil, lymphocyte, and platelet counts are generally easily lost in clinical practice, which could have added selection bias to the outcomes. Finally, nonlinear models must be used in future research to examine the relationships between blood Pb and SII.

## 5. Conclusions

In this study, we found that Mn, Cd and co-exposure to Mn and Cd could affect the systemic immune-inflammatory condition in humans. Our findings also showed that the immune-inflammatory response could be aggravated in a linear dose-dependent manner by Mn and Cd as well as co-exposure to Mn and Cd. However, neither blood Pb nor its co-exposure to other metals had a similar impact on the immune-inflammatory response. Further prospective research is still required to validate these findings.

## Figures and Tables

**Table 1:** The distribution of blood Mn, Cd and Pb concentrations according to baseline characteristics of the study participants.

Characters	Participants [n (%)]	Blood Mn (μg/L)	*p* Value	Blood Cd (μg/L)	*p* Value	Blood Pb (μg/dL)	*p* Value
**Age**							
20–49	1065 (49.0)	9.84 (7.92, 12.53)	**<0.001**	0.25 (0.16, 0.44)	**<0.001**	0.68 (0.47, 1.11)	**<0.001**
49–65	618 (28.4)	9.21 (7.55, 11.60)		0.33 (0.20, 0.65)		1.18 (0.82, 1.82)	
>65	491 (22.6)	8.85 (7.01, 10.80)		0.35 (0.24, 0.56)		1.43 (0.94, 2.14)	
**Sex**							
Male	1065 (49.0)	8.77 (7.18, 13.52)	**<0.001**	0.27 (0.17, 0.49)	**<0.001**	1.15 (0.72, 1.84)	**<0.001**
Female	1109 (51.0)	10.03 (8.17, 12.72)		0.33 (0.21, 0.55)		0.81 (0.52, 1.33)	
**Race/ethnicity**							
Mexican American	662 (30.5)	9.93 (7.92, 12.54)	**<0.001**	0.26 (0.18, 0.42)	**<0.001**	0.89 (0.57, 1.41)	**<0.001**
Non-Hispanic white	778 (35.8)	9.03 (7.51, 11.30)		0.29 (0.17, 0.54)		0.96 (0.58, 1.66)	
Non-Hispanic black	424 (19.5)	8.36 (6.76, 12.39)		0.34 (0.20, 0.66)		1.01 (0.60, 1.68)	
Others	310 (14.3)	11.24 (9.00, 14.33)		0.36 (0.24, 0.62)		1.11 (0.69, 1.65)	
**Education levels**							
No and elementary school	253 (11.6)	9.98 (7.73, 12.27)	0.136	0.30 (0.20, 0.56)	**<0.001**	1.16 (0.74, 1.92)	**<0.001**
Middle school	236 (10.9)	9.66 (7.86, 11.77)		0.40 (0.24, 0.71)		1.20 (0.76, 1.88)	
High school	483 (22.2)	9.22 (7.34, 11.75)		0.34 (0.21, 0.66)		0.34 (0.58, 1.65)	
College or over	1201 (55.3)	9.34 (7.63, 11.84)		0.27 (0.17, 0.44)		0.27 (0.56, 1.42)	
**Annual house income**							
<20,000	2059 (94.7)	9.41 (7.83, 11.63)	0.726	0.30 (0.18, 0.53)	**0.018**	0.95 (0.42, 1.56)	0.183
>20,000	115 (5.3)	9.43 (7.55, 12.91)		0.26 (0.17, 0.38)		1.08 (0.63, 1.70)	
**BMI**							
<25	578 (26.6)	9.43 (7.71, 11.97)	**0.024**	0.35 (0.19, 0.66)	**<0.001**	1.07 (0.64, 1.76)	**<0.001**
25–30	701 (32.2)	9.21 (7.30, 11.60)		0.29 (0.19, 0.50)		1.09 (0.69, 1.72)	
≥30	895 (41.2)	9.53 (7.85, 11.07)		0.27 (0.18, 0.47)		0.83 (0.54, 1.33)	
**Alcohol intake**							
Yes	15,264 (70.1)	9.19 (7.52, 11.64)	**<0.001**	0.30 (0.18, 0.54)	0.260	1.01 (0.62, 1.64)	**<0.001**
No	650 (29.9)	9.96 (7.90, 11.47)		0.29 (0.18, 0.49)		0.87 (0.56, 1.45)	
**Exposure to smoking**							
Yes	1394 (64.1)	9.31 (7.60, 11.75)	0.176	0.34 (0.20, 0.67)	**<0.001**	1.04 (0.62, 1.69)	**<0.001**
No	780 (35.9)	9.57 (7.71, 12.02)		0.25 (0.17, 0.37)		0.87 (0.57, 1.40)	
**SII**							
<357.52	724 (33.3)	9.14 (7.31, 11.58)	**<0.001**	0.29 (0.18, 0.48)	0.264	1.01 (0.62, 1.55)	0.556
357.52–554.91	726 (33.4)	9.41 (7.62, 11.63)		0.29 (0.19, 0.51)		0.95 (0.59, 1.51)	
>554.91	724 (33.3)	9.71 (7.87, 12.46)		0.30 (0.19, 0.57)		0.92 (0.58, 1.62)	

Note: Data were presented as median (interquartile range) or number (%). Abbreviations: BMI, body mass index; SII, systemic immune-inflammation index.

**Table 2:** Spearman correlations of Mn, Cd and Pb with SII, age and BMI.

Variables	Mn	*p* Value	Cd	*p* Value	Pb	*p* Value
SII	0.075	0.001 **	0.055	0.011 *	0.090	0.629
Age	−0.144	<0.001 ***	0.076	<0.001 ***	0.286	<0.001 ***
BMI	0.045	0.037 *	−0.093	<0.001 ***	−0.137	<0.001 ***

* *p* < 0.05, ** *p* < 0.01, *** *p* < 0.001. Abbreviations: BMI, body mass index; SII, systemic immune-inflammation index.

**Table 3:** Association of SII with blood Mn, Cd and Pb levels in the study population.

Variables	*β* (95% CI), *p* Values for SII		
	Crude Model	Mode I ^a^	Model Ⅱ ^b^
Blood Mn (μg/L)			
Continuous ^c^	5.80 (2.53, 9.06) 0.001	5.65 (2.33, 8.97) 0.001	5.96 (2.62, 9.29) <0.001
T1 (<8.21)	0 [Reference]	0 [Reference]	0 [Reference]
T2 (8.21–10.94)	−7.45 (−37.85, 22.96) 0.631	−11.42 (−41.85, 19.00) 0.462	−10.13 (−40.56, 20.31) 0.514
T3 (>10.94)	36.57 (5.95, 67.18) 0.019	32.67 (1.64, 63.70) 0.039	33.49 (4.31, 66.66) 0.026
*p* value for trend	0.021	0.040	0.027
Blood Cd (μg/L)			
Continuous c	30.06 (7.00, 53.12) 0.011	39.88 (16.74,63.03) 0.001	42.10 (18.05, 66.14) 0.001
T1 (<0.22)	0 [Reference]	0 [Reference]	0 [Reference]
T2 (0.22–0.42)	14.20 (−16.49, 44.90) 0.364	14.32 (−16.73, 45.34) 0.336	15.20 (−16.00, 46.40) 0.340
T3 (>0.42)	30.80 (0.57,61.03) 0.046	45.16 (13.78,76.55) 0.005	48.91 (16.20,81.64) 0.003
*p* value for trend	0.006	0.005	0.004
Blood Pb (μg/dL)			
Continuous c	2.02 (−7.99, 12.03) 0.692	6.42 (−74.20, 17.04) 0.236	6.63 (−4.13, 17.40) 0.227
T1 (<0.71)	0 [Reference]	0 [Reference]	0 [Reference]
T2 (0.71–1.33)	−20.93 (−51.47, 9.61) 0.179	−16.64 (−48.91, 15.63) 0.312	−17.93 (−50.93, 14.52) 0.279
T3 (>1.33)	−13.76 (−44.46, 16.94) 0.380	0.62 (−34.94, 36.17) 0.973	−1.31 (−37.63, 35.00) 0.943
*p* value for trend	0.378	0.936	0.979

^a^ Model I was adjusted for age, sex, race/ethnicity and BMI. ^b^ Model Ⅱ was adjusted for age, sex, race/ethnicity, BMI, education, annual family income, alcohol intake and exposure to smoking. ^c^ SII was a continuous variable.

**Table 4:** Significant associations of SII with co-exposure to Mn and Cd in the study population.

Variables	*β* (95% CI), *p* Values for SII		
	Crude Model	Model I ^a^	Model Ⅱ ^b^
Mn*Cd			
Continuous ^c^	2.76 (0.86, 4.66) 0.004	3.59 (1.58, 5.39) <0.001	3.66 (1.69, 5.60) <0.001
T1 (<2.00)	0 [Reference]	0 [Reference]	0 [Reference]
T2 (2.00–4.24)	21.70 (−8.88, 52.28) 0.164	20.77 (−9.83, 51.37) 0.183	22.22 (−8.58, 53.02) 0.157
T3 (>4.24)	47.85 (17.29, 78.41) 0.002	53.34 (27.08, 89.60) <0.001	62.57 (30.31, 94.82) <0.001
*p* value for trend	0.002	<0.001	<0.001

^a^ Model Ⅰ was adjusted for age, sex, race/ethnicity and BMI. ^b^ Model Ⅱ was adjusted for age, sex, race/ethnicity, BMI, education, annual family income, alcohol intake and exposure to smoking. ^c^ SII was a continuous variable.

## Data Availability

The data are publicly available on the NHANES website: https://www.cdc.gov/nchs/nhanes/Index.htm (accessed on 10 March 2023).
